# Trigeminal neuralgia associated with dural arteriovenous fistula: a case report and literature reviews

**DOI:** 10.3389/fneur.2023.1293056

**Published:** 2023-12-07

**Authors:** Hao Zhou, Xingrong Wei, Defeng Zeng, Shuguang Zhang, Xueqian Hu, Zhenqin Wei, Yang Li

**Affiliations:** ^1^Department of Neurosurgery, The First Affiliated Hospital of Dalian Medical University, Dalian, China; ^2^Department of Graduate School, Dalian Medical University, Dalian, China

**Keywords:** trigeminal neuralgia, dural arteriovenous fistula, case report, embolization, microvascular decompression

## Abstract

Trigeminal neuralgia is a paroxysmal, intense electric shock-like, or knife-like, recurrent pain that affects one or more sense areas of the unilateral facial trigeminal nerve. It can be classified into two groups from an etiological standpoint: primary and secondary. The pain episodes brought on by such vascular compression are still categorized as primary trigeminal neuralgia, despite the fact that microvascular compression of the trigeminal nerve root has now been demonstrated to be the primary cause. A rare and complicated condition known as a dural arteriovenous fistula (DAVF) can irritate the Gasserian ganglion or compress the trigeminal nerve’s root entry zone (REZ), leading to secondary trigeminal neuralgia (TN). At present, the treatment of DAVF-induced trigeminal neuralgia is not conclusive. This article reports a case of DAVF-induced trigeminal neuralgia cured by MVD and reviews the relevant literature.

## 1 Introduction

Trigeminal neuralgia (TN) is a severe form of facial pain that can be associated with a significant reduction in quality of life. The peak age of onset for TN is between 50 and 70 years old, with most cases affecting middle-aged and elderly people. With a ratio of 1.4 to 1, women experience this condition more frequently than men. With a ratio of approximately 1.2:1, the right side of the face is typically more affected than the left. TN usually results from compression of the trigeminal nerve entry zone by an aberrant loop of the artery or vein, especially the superior cerebellar artery (75% of cases) and anteroinferior cerebellar artery (10% of cases). Much less frequently, posterior fossa lesions such as neoplasms, aneurysms, vascular malformations, or vertebrobasilar artery ectasia may compress the trigeminal nerve entry zone and result in secondary TN. Dural arteriovenous fistulas as a cause of TN are extremely rare with less than 20 previously reported cases.

Trigeminal neuralgia can be classified into two types. Abnormal blood vessels compress the ventral side of the nerve’s root entry zone (REZ), resulting in ischemia of branches II and III in the walking area of the trigeminal nerve. This leads to demyelination changes of Aβ and Aδ and the formation of false synapses between fibers. As a result, even slight stimulation of the facial area can cause cross-activation of Aβ and Aδ, resulting in the characteristic shock-like pain, which is type 1 trigeminal neuralgia. Type 2 TN is characterized by dull pain that manifests as burning sensations in the cheek as well as intermittent dull pain. The etiology of this type of neuralgia is complex, and the specific mechanism is not yet clear. It is speculated that the condition is closely related to Class C fibers. It is important to note that type 1 and type 2 TN are not distinct pathologic processes, but rather variations of the same disease. Type 1 TN patients tend to be older, have right-sided disease, have a shorter course of disease, are primarily affected by arterial compression, are relieved immediately after MVD, and have a very low recurrence rate within 2 years when compared to type 2 TN patients. The type of the initial pain is more useful in classifying the disease than post-hospital assessment, even though a small percentage of patients gradually transition from type 1 to type 2 TN. As a result, the prognosis is still similar to that of type 1 TN.

Dural arteriovenous fistula (DAVF) is also known as dural arteriovenous fistula-like vascular malformation. The supplying arteries drain through the orifice fistula located in the dural membrane to the meningovenous sinus, or to the cortical or deep veins, which cause eddy currents and high pressure in the venous sinus and reflux to the adjacent bridging veins. The latter causes increased intracerebral venous pressure, reflux obstruction, tortuosity, and even rupture bleeding. According to the location of the fistula, there were cavernous sinuses (33.3%), tentorial cerebellum (25.9%), transverse, sigmoid or sinuses (13.0%), anterior skull base (11.1%), superior sagittal sinus (11.1%), and others (5.6%). Borden classified the fistula into type I (29.6%), type II (29.6%), and type III (40.7%). The anterior skull base, tentorial cerebellum, and craniocervical junction were mainly Borden III type. The superior sagittal sinus, transversal sinus, sigmoid sinus, or sinuses were mostly of type II, and there were also type I or type III, and the most common combination was distal venous sinus stenosis or occlusion. DAVFs in cavernous sinus were mostly type I. Type I has a good prognosis, with very few intracranial hemorrhage or neurological dysfunction (2%); in type II, 38–40% of patients have intracranial hemorrhage or neurological dysfunction; in type III, there is a great chance of hemorrhage (79–100%), and the prognosis is poor. Therefore, Borden type II and type III patients need treatment, especially those with tortuous drainage veins and tumor-like dilatation, need to be treated as soon as possible to prevent rupture and bleeding. Whether Borden type I cases should be treated is controversial. In addition, 227 patients in Finland were followed up for a median of 10 years, and it was found that DAVF in the transverse sinus and sigmoid sinus had little effect on the survival of patients, while those with other sites or cortical venous reflux had significant effect.

## 2 Case report

A 58-year-old man, previously healthy, presented with recurrent pain in his left face in the V3 distribution for 5 years. The pain was explained as intermittent, sharp, and shock, triggered by chewing or touching his face. Approximately 2 years ago, he was diagnosed as TN in another hospital and began to take carbamazepine orally. However, the symptom aggravated and even high-dose carbamazepine could not control the pain effectively. The patient had no history of pulsatile tinnitus or facial numbness. Neurologic examination revealed no abnormal findings.

**Figure 1:**
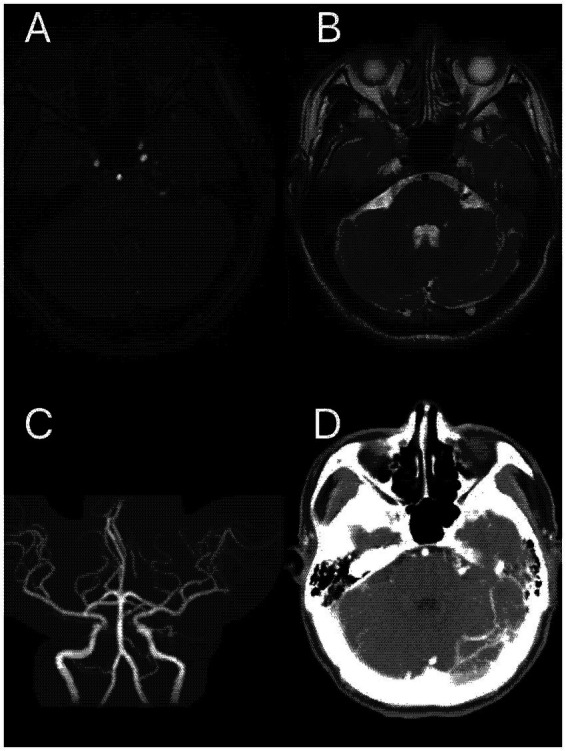
A malformed vascular mass in the left pontine cerebellar horn region seen on MRI scanning **(A,B)**; Panel **(C)** is magnetic resonance angiography (MRA); and Panel **(D)** is unreconstructed CTA showing a clear vascular malformation.

**Figure 2:**
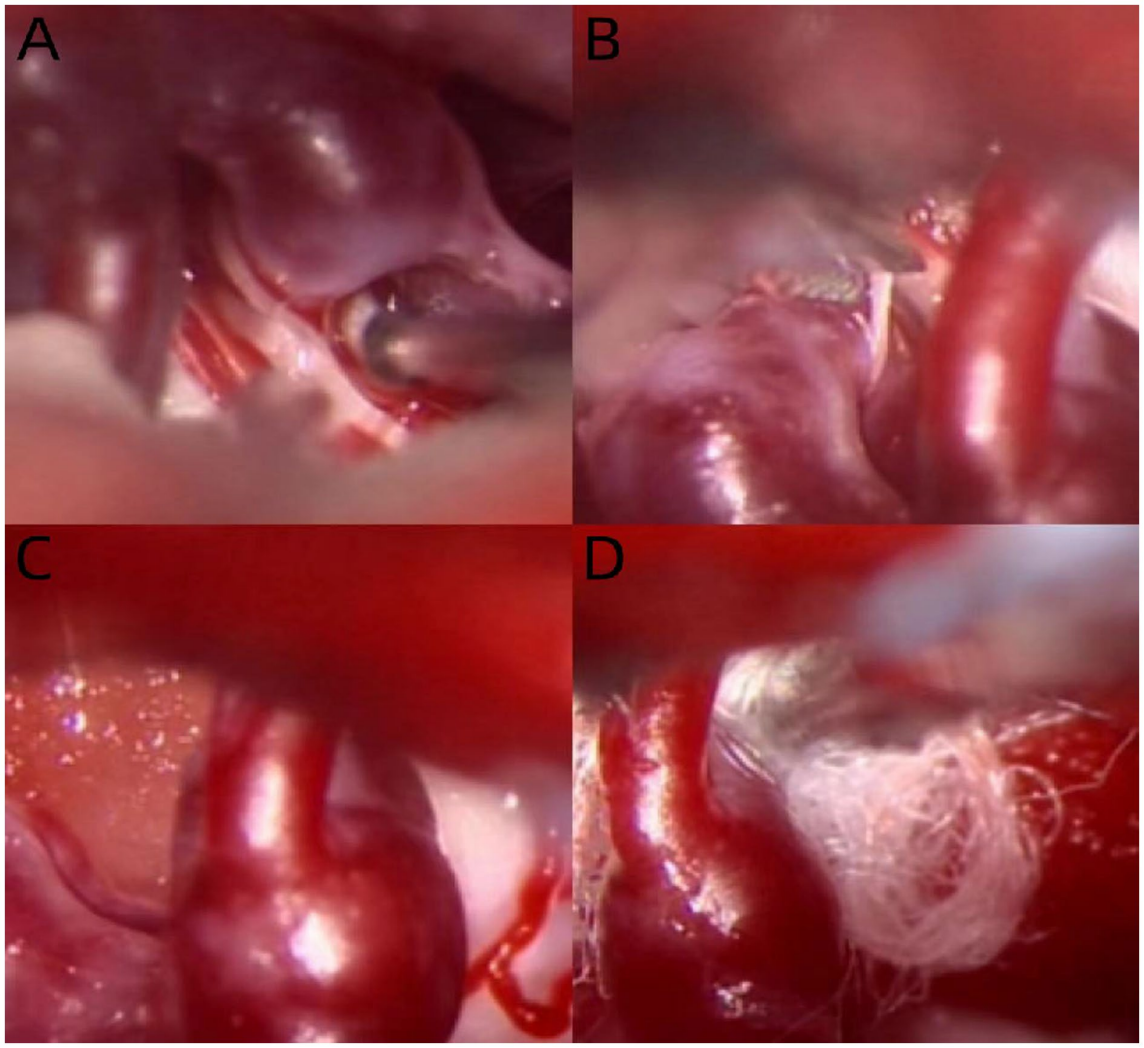
Intraoperative pictures of patients in case report: the malformed vascular mass compresses the left trigeminal nerve and passes between the motor branches of the trigeminal nerve **(A)**; sever the motor branch of the trigeminal nerve and free the malformed blood vessels **(B)**; then the malformed blood vessels are completely free **(C)**; and a gesket is inserted between the malformed vascular mass and the trigeminal nerve **(D)**.

**Figure 3:**
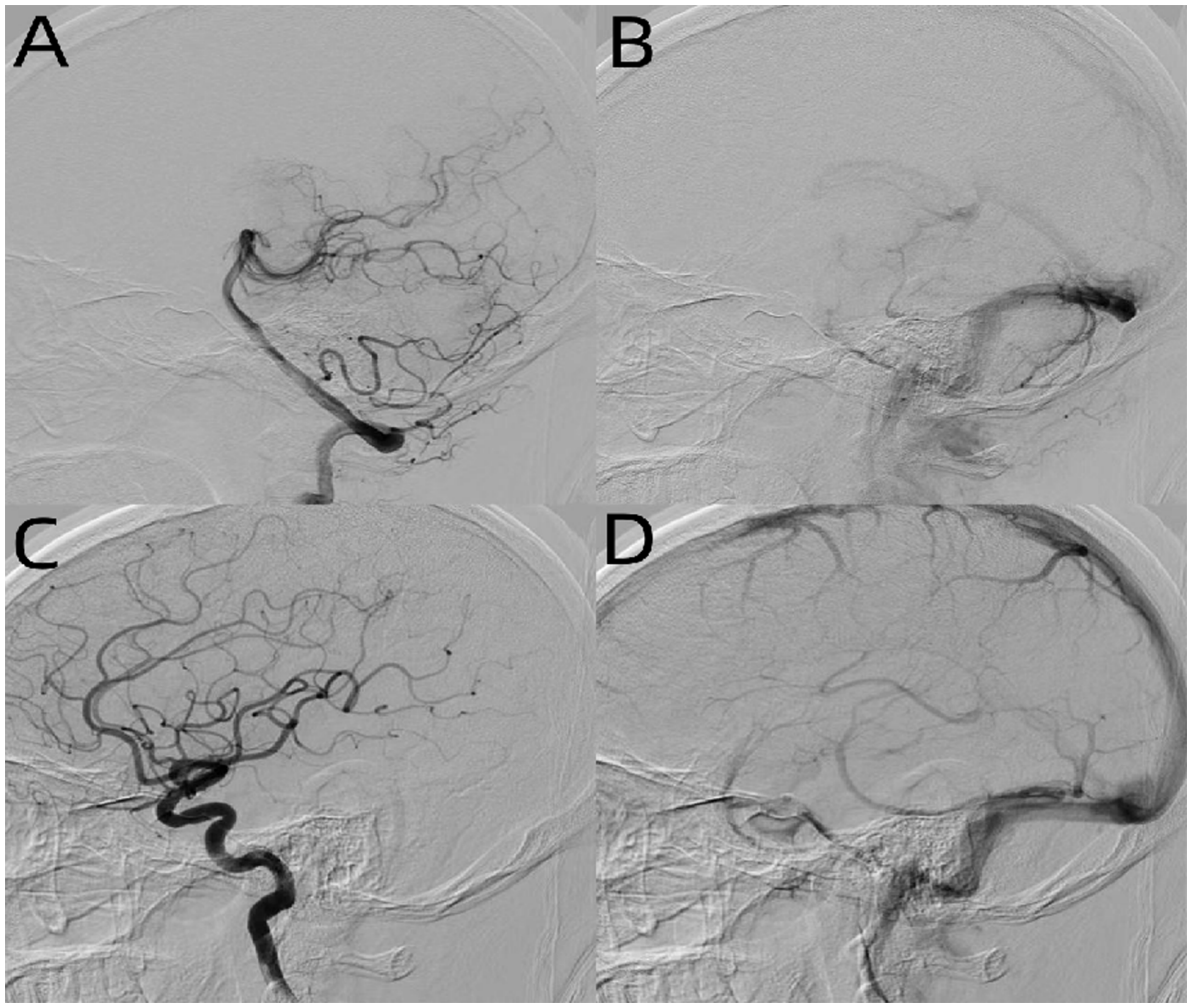
DSA of patients after MVD in case report: left vertebral arterial phase **(A)**, left internal carotid system arterial phase **(C),** and venous phase **(B,D)** found no obvious abnormality.

A curved mass of blood vessels was discovered close to the left trigeminal nerve in the brain during MRI and MRA scans (Figure 1). Following the head and neck combined with CTA, it was discovered that the left cerebellar corner area had a curved venous malformation, with the vein draining into the rectus sinus. The patient and his family were categorized as having Borden type I dural arteriovenous fistula. Considering the patient’s age is not old, the bleeding risk of the dural arteriovenous fistula is low, the intervention may embolize the draining vein of the brainstem, which may cause more serious consequences, and the trigeminal neuralgia is not necessarily resolved; combined with the opinions of the patient and his family, microvascular decompression surgery was finally chosen, in which the malformed vascular mass in the left side of the pontine angle area was seen to compress the trigeminal nerve, and the vasculature and nerves were separated, and two rocky veins were electrocoagulated and disconnected, which were close to the trigeminal nerve (Figure 2). The patient was discharged from the hospital after recovering well. After 6 months, the patient was followed up and underwent total cerebral angiography, but no obvious abnormality was found (Figure 3), which was beyond our expectation, and this might be related to the fact that the rock vein was dissected during the operation and the dural arteriovenous fistula was not serious.

## 3 Literature review

The subject words and free words of “trigeminal neuralgia” and “dural arteriovenous fistula” were used to search the two major databases of PubMed and Embase, and a total of 68 literature studies were retrieved. Through reading, 14 cases of dural arteriovenous fistula-caused trigeminal neuralgia were selected, including 15 cases. Patient characteristics, such as sex and age, any medication use and duration of failure, distribution of TN symptoms, any additional symptoms, description of DAVF, surgery performed throughout the course of the disease, clinical and radiological findings, duration of follow-up, and any adverse events, were collected and tabulated. We chose to use the Borden classification system to classify DAVFs because it is less complex and can be more easily applied to case populations with varying levels of descriptive detail (Table 1). When an article provides a Cognard type, convert it to a Borden class using the following scheme: Borden class 1 is equivalent to Cognard class 1 or IIa, Borden class 2 is equivalent to Cognard Class IIb or iatsr b, Borden class 3 is equivalent to Cognard class III, IV, or V. When the article does not provide Borden or Cognard classification, we apply Borden class 1, and if the picture of the article is clear enough, we make the Borden classification based on the provided image description.

**Table 1:** Patients with dural arteriovenous fistulas (DAVF) presenting as trigeminal neuralgia reported in literature.

Author(s), Year	Patient (n) and Sex	Age (years)	Borden type	Time to medication failure (Medication)	TN type (Division)	Additional symptoms	Compression site	Procedures	Pain relief	Radiographic outcome	Adverse events
Ahmed et al. (3)	1, M	56	I	N/A (carbamazepine and lamotrigine)	TN 1 (V3)	None	GG	Embolisation	Significant relief, 1 year	N/S	None
Akhaddar et al. (4)	1, M	42	I	N/A	TN 1 (V3)	None	REZ	Coagulat and divid draining vein	Complete relief	N/S	None
Brown et al. (5)	1, M	55	III	N/A	TN1 then rapidly TN2(V2and V3)	None	REZ	embolisation	Partial relief, complete relief after 4 months	Complete occlusion	None
	1, F	72	I	N/A	TN1 for 20 years, then TN2 (V3)	Hearing loss	REZ	MVD	Poor effect, Trigemi- nal tractotomy can be considered	N/S	None
Chen et al. (6)	1, M	35	III	N/A	TN 1 (V2 and V3)	None	REZ	Disconnect hunt point and draining vein	Complete relief	Disappeared and no recanalization	None
Du et al. (7)	1, F	77	I	N/A(gabapentin and acetaminophen/hydrocodone)	TN 1 (V3)	None	GG	Embolisation	Complete relief	Complete occlusion	Subarachnoid hemorrhage hydrocephalus
Fukutome et al. (8)	1, F	75	I	N/A	TN 2 (V1 and V2)	None	GG	Embolisation	Complete relief	Complete occlusion	None
Ghodasara et al. (9)	1, M	60	I	N/A	TN 1 (V2 and V3)	Walk difficulty	REZ	ClipandMVD	Complete relief	Complete occlusion	None
Jiang et al. (10)	1, M	57	I	2 years (carbamazepine)	TN 1 (V2 and V3)	None	REZ	Embolisation and resection of the embolized dilated vessels	Complete relief	Complete occlusion	None
Lu et al. (11)	1, M	58	III	2.5 years (carbamazepine)	TN 1 (V2 and V3)	None	C REZ	Embolisation	Complete relief	Complete occlusion	None
de Paula Lucas and Zabramski (12)	1, M	50	III	N/A	TN 1 (V3)	Pulsatile tinnitus	REZ	Resection	Complete relief	Disappeared and no recanalization	None
Mendes et al. (13)	1, F	53	I	A month (carbamazepine)	TN 1 (V1)	None	REZ	embolisation	Complete relief	complete occlusion	None
Mulcahy et al. (14)	1, M	51	III	N/A	TN 1 (V1)	Conjunctival injection and tearing	REZ	Embolisation	Complete relief	Complete occlusion	None
Okromelidze et al. (15)	1, F	71	II	N/A(carbamazepine)	TN 1 (V2 and V3)	None	REZ&GG	Gamma knife radiosurgery	Complete relief	Caliber of the vessels decreased	None
Matsushige et al. (16)	1, M	50	II	N/A	TN 1 (V2 and V3)	None	REZ	Gamma knife radiosurgery	Partial relief, complete relief after carbamazepine for 1 year	Thrombosed lesion 1 year after GKS	None

Among the 15 patients, seven cases were completely occluded by means of intervention to treat dural arteriovenous fistula, and the trigeminal neuralgia was well relieved or cured after surgery. However, one case underwent ventriculoperitoneal shillage due to postoperative subarachnoid hemorrhage and hydrocephalus, and the postoperative recovery was good, and the trigeminal neuralgia did not relapse. In two cases, craniotomy was performed, the lesion was blocked or resected without microvascular decompression, and trigeminal neuralgia was relieved. The other case was treated with an interventional craniotomy to cure trigeminal neuralgia without microvascular decompression; there were also two patients underwent microvascular decompression, of which one patient only underwent microvascular decompression of the trigeminal nerve, and the postoperative symptoms were not significantly relieved compared with the previous one; the other patient underwent the combination of arterial vein clamping and microvascular decompression of the fifth cranial nerve; the postoperative symptoms were improved and relieved, and the prognosis was good. Finally, two cases were treated with dural static and static fistulas by gamma knife stereotactic radiotherapy, and the symptoms of the patients were relieved slowly after surgery. However, after 6 months to 1 year of follow-up, the symptoms of trigeminal neuralgia were significantly relieved and no other discomfort was found.

## 4 Discussion

Dural arteriovenous fistula is a rare cause of trigeminal neuralgia, which is usually caused by dural arteriovenous fistula in the transverse sinus of the posterior fossa or sigmoid sinus compression of the fifth cranial nerve REZ region or by dural arteriovenous fistula compression GG in the cavernous sinus region. Its pain properties and characteristics are consistent with primary trigeminal neuralgia, so preoperative magnetic resonance examination of the head is particularly important (1). Once the malformed blood vessels are found, total cerebral angiography is necessary to confirm the diagnosis and make the next diagnosis and treatment plan. Among the 15 patients recruited in this study, most of them simply treated dural arteriovenous fistula through intravascular intervention, and the postoperative symptoms of trigeminal neuralgia were well relieved, which is also the mainstream way to deal with such diseases at present. It has the advantages of less trauma and exact efficacy and at the same time solves the risk of bleeding in the later period of dural arteriovenous fistula. We reported a case of trigeminal neuralgia caused by a dural arteriovenous fistula. Due to complex vascular malformations involving return veins of the cerebellum and brainstem, simple treatment of vascular malformations was too risky, so only microvascular decompression and electrocoagulation of part of small veins were performed, and the patient’s symptoms of trigeminal neuralgia completely disappeared after surgery with no other discomfort. Dural arteriovenous malformations in the cavernous sinus mentioned above are generally type I with low bleeding risk, while dural arteriovenous fistulas in the transverse sinus of the posterior fossa or sigmoid sinus are usually type II with little impact on patients’ survival. For a class of dural arteriovenous fistula-induced trigeminal neuralgia with Borden I and II, no other obvious clinical symptoms (such as intracranial noise, headache, vision loss, and hypoperfusion), complicated intravascular intervention and poor prognosis, the traditional microvascular decompression can still be used to solve the problem of trigeminal neuralgia, and the primary dural arteriovenous fistulas can be followed up. MVD or interventional embolization should be taken into consideration in conjunction with the patient’s unique circumstances when treating trigeminal neuralgia brought on by DAVF (2). We consider that MVD should be the first option for treating trigeminal neuralgia brought on by Borden type I low-flow DAVF, first of all, through micromanipulation, MVD can more effectively relieve the pressure of malformed blood vessels on the trigeminal nerve, relieve pain, and reduce the recurrence rate. Second, MVD can lessen the chance of postoperative brain tissue edema and bleeding while avoiding the effects on intracranial venous return. Despite the risk of serious complications, such as intracranial hemorrhage, infection, cerebellar infarction, and even death, it is sufficient for more long-term benefits in younger patients. For older patients, radiofrequency ablation of extracranial non-Gasserian, which is safer and more selective for branches, should also be a good option. Trigeminal neuralgia caused by dural arteriovenous fistula is relatively rare, and there are more factors to be considered for the choice of procedure, and we need more cases to be analyzed and summarized.

## Data availability statement

The original contributions presented in the study are included in the article/supplementary material; further inquiries can be directed to the corresponding authors.

## Ethics statement

Written informed consent was obtained from the individual(s) for the publication of any potentially identifiable images or data included in this article.

## Author contributions

HZ: Conceptualization, Data curation, Investigation, Methodology, Project administration, Software, Writing – original draft. XW: Funding acquisition, Investigation, Methodology, Resources, Supervision, Visualization, Writing – original draft. DZ: Data curation, Formal analysis, Resources, Investigation, Writing – review & editing. SZ: Data curation, Formal analysis, Resources. XH: Data curation, Investigation. ZW: Funding acquisition, Resources, Writing – review & editing. YL: Funding acquisition, Resources, Writing – review & editing.
